# Weight management barriers and facilitators after breast cancer in Australian women: a national survey

**DOI:** 10.1186/s12905-020-01002-9

**Published:** 2020-07-06

**Authors:** Carolyn Ee, Adele Elizabeth Cave, Dhevaksha Naidoo, Kellie Bilinski, John Boyages

**Affiliations:** 1 NICM Health Research Institute, Western Sydney University, Locked Bag 1797, Penrith, NSW Australia; 2 ICON Cancer Centre, Sydney Adventist Hospital, Wahroonga, NSW 2076 Australia

**Keywords:** Breast cancer, DCIS, Obesity, Weight gain, Barriers and facilitators, Lifestyle

## Abstract

**Background:**

Breast cancer is the most common cancer in women worldwide. Weight gain after breast cancer is associated with poorer health outcomes. The aim of this study was to describe how Australian breast cancer survivors are currently managing their weight.

**Methods:**

Online cross-sectional survey open to any woman living in Australia who self-identified as having breast cancer, between November 2017 and January 2018.

**Results:**

We received 309 responses. Most respondents described their diet as good/excellent and reported moderate-high levels of weight self-efficacy. Despite this, the proportion of overweight/obesity increased from 47% at time of diagnosis to 67% at time of survey. More than three quarters of respondents did not receive any advice on weight gain prevention at the time of diagnosis. 39% of women reported being less active after cancer diagnosis, and and few weight loss interventions were perceived to be effective. Facilitators were structured exercise programs, prescribed diets, and accountability to someone else, while commonly cited barriers were lack of motivation/willpower, fatigue, and difficulty maintaining weight. Women who cited fatigue as a barrier were almost twice as likely to be doing low levels of physical activity (PA) or no PA than women who did not cite fatigue as a barrier.

**Conclusions:**

We report high levels of concern about weight gain after BC and significant gaps in service provision around weight gain prevention and weight management. Women with BC should be provided with support for weight gain prevention in the early survivorship phase, which should include structured PA and dietary changes in combination with behavioural change and social support. Weight gain prevention or weight loss programs should address barriers such as fatigue. More research is required on the effectiveness of diet and exercise interventions in BC survivors, particularly with regard to weight gain prevention.

## Background

Globally, breast cancer is the most common cancer in women [1–3]. There were over 2 million new diagnoses of breast cancer (BC) worldwide in 2018, with this figure expected to rise to 3 million by 2040 [2]. Obesity is a known risk factor for BC [4] and may lead to poorer outcomes for BC survivors. A meta-analysis of 82 studies reported a 41% relative increase in all-cause mortality for breast cancer survivors with obesity compared with women of normal weight, with a higher risk in premenopausal women [5].

Additionally, weight gain after breast cancer is common [3] and may increase the risk of disease recurrence and mortality. A meta-analysis of eight studies, including observational studies and randomised controlled trials, reported that weight gain of greater than 10% of baseline body weight was associated with a hazard ratio of 1.23 for all-cause mortality compared with weight maintenance, and may be associated with an increase in BC recurrence [6]. Weight gain after breast cancer diagnosis is thought to be multifactorial and related to the use of systemic treatment as well as changes in lifestyle [3]. Given the growing population of breast cancer survivors, increased survival due to advances in treatment [7] and the link between weight gain and adverse health outcomes, research into weight after breast cancer is of critical importance.

It is anticipated that there will be 25,000 new cases of BC diagnosed annually in Australia by 2040 [1]. Yet, there is a relative paucity of research addressing the needs of women who experience weight gain in Australia. One prospective cohort study described the changes in weight gain in women diagnosed with early breast cancer in the state of Queensland [8], however there has not been any national population-based data until the publication of our national survey in 2020 [9]. Moreover, there is a lack of research about barriers and facilitators of weight management after breast cancer in Australia. A qualitative study of 14 women with BC who had been randomized to a 12-month weight loss intervention explored women’s experiences of making weight, dietary and physical activity (PA) changes during the trial [10], however little is known about barriers and facilitators of weight management in real-world conditions as opposed to weight management within the context of a clinical trial.

The aim of this study was to describe the management of weight amongst respondents to a cross-sectional Australian survey and explore barriers and facilitators of successful weight management in this population.

## Methods

### Study design and inclusion criteria

Our methods have been previously described [9]. We conducted an anonymous self-administered online cross-sectional survey from November 2017 until January 2018 using the survey platform Qualtrics [11]. Women who -self-identified as having breast cancer and who were living in Australia were invited to complete the survey. Women were recruited from the Breast Cancer Network Australia (BCNA) Review and Survey Group, who have agreed to receive emails about research studies. BCNA is the largest breast cancer advocacy group in Australia. BCNA have decided to limit research requests to this select group, therefore allowing researchers to access women who are engaged in the research process, while protecting the rest of BCNA from frequent research requests. The survey was emailed on December 5th, 2017 and a reminder email was sent to 1835 members on January 15th, 2018 (Additional file 1). We also recruited women from online communities (women’s health organization social media pages, online breast cancer support groups in Australia) and through word of mouth.

### Survey instrument

Two clinicians (CE, a general practitioner/family physician and JB, a radiation oncologist) developed the survey after reviewing previous literature on weight after BC and incorporated feedback from six BCNA representatives and several health researchers. The 60-item survey included questions on the characteristics, medical details such as diagnosis and treatment, lifestyle habits, and weight and weight management of women. Ethics approval for this study was provided by the Human Research Ethics Committee, Western Sydney University (H12444, Oct 2017). Additional file 1 contains details of the specific demographic, medical, menopausal and lymphoedema data that were collected in the survey. In this manuscript we report on how women were managing their weight, and the perceived barriers and facilitators to successful weight management.

### Weight after diagnosis

Weight was self-reported by the survey respondents, who were asked about their current weight (kg) and height (m) at time of diagnosis. Body Mass Index was calculated from these measures as weight/height^2^. A Pearsons correlation was performed to test the relationship between weight gain and time since diagnosis. Women were asked about the pattern of weight since diagnosis with options for “gained weight overall”, “lost weight overall”, “weight stable” or “weight has fluctuated a great deal”. We used an 11-point Likert scale to assess concern about weight from 0 (not at all concerned) to 10 (very concerned). Experiences with a range of weight loss interventions and the perceived effectiveness of the interventions on was described using a five-point Likert scale from 1 (not at all effective) to 5 (very effective). The responses were further dichotomized into 1 to 2 (not effective) and 3 to 5 (effective). Women were also asked about perceived barriers and facilitators to successful weight loss and weight maintenance, and what they believed should be research priorities in this area.

### Lifestyle habits

Women were asked about any specific diets followed, intake of recommended daily serves of fruit and vegetables, advice received as to restricting diet, self-assessed diet quality on a five-point Likert scale from 1 (poor) to 5 (excellent), cigarette use, alcohol use, self-assessed PA level, and self-assessed health status. The validated Weight Self Efficacy Scale (WEL-SF) [12] was used to evaluate how confident women now felt about being able to successfully resist the desire to overeat in eight different situations on an 11-point Likert scale from 0 (not confident at all) to 10 (very confident). We further dichotomised the responses into “Not confident” (0–4) and “Confident” (5–10). PA levels were calculated according to the number of 20-min sessions of less vigorous exercise or more vigorous exercise a week, given a weighting and described in terms of MET (metabolic cost) minutes where MET minutes less than 80 were coded as no PA, 80 to 400 as low, 400 to 560 as moderate and more than 560 as high. A value of 4 METs was given to moderate PA and 7.5 to vigorous PA [13].

### Statistical analysis

Stata Corp 13.1 [14] was used to analyse the data presented in this report and the data analysis used descriptive statistics, as well as odds ratio analyses to explore associations between medical symptoms, cited barriers, and lifestyle habits.

## Results

### Survey response

The response rate from the BCNA Review and Survey group was 15% (283/1857). A further 26 women responded to the survey from other channels giving a total of 309 responses, of which 273 completed the survey (95.8% completion rate).

### Sample characteristics

Our sample has been previously described [9]. Table 1 describes the demographic characteristics of respondents. The majority of women were Caucasian (92.5%, *n* = 285) with a mean age of 59.1 years (*SD* = 9.5, range 33–78, *n* = 298). Characteristics were similar across BCNA members and non-BCNA respondents except that there was a higher proportion of women in the non-BCNA group who were self-employed (23% vs 10%) and in the BCNA group who were retired (33% vs 23%), although there were no differences between these groups on Pearson’s Chi-squared test, *X*^*2*^ (7, *N* = 308) = 6.9912, *p* = 0.430. The majority of women (83%) had been diagnosed with Stage 0-III breast cancer. The mean time since diagnosis of breast cancer was 8.22 years (*S.D* = 5.14, range = 1–32 years). Most women were either premenopausal (43%) or perimenopausal (12%) at the time of diagnosis.

**Table 1:** Demographic characteristics of survey respondents

	Description	N (responses)	Percentage
**State (*n*** **= 309)**			
	Australian Capital Territory	14	4.5
	New South Wales	91	29.5
	Northern Territory	0	0.0
	Queensland	48	15.5
	South Australia	28	9.1
	Tasmania	3	1.0
	Victoria	95	30.7
	Western Australia	30	9.7
**Education (*n*** **= 307)**			
	High school- year 10	30	9.8
	High school- year 12	35	11.4
	Vocational College	55	17.9
	Bachelor’s degree	90	29.3
	Postgraduate degree	97	31.6
**Ethnicity (*n*** **= 308)**			
	European/Anglo Saxon/Caucasian	285	92.5
	Asian	5	1.6
	Oceanic (incl. Australian and New Zealand first peoples, Polynesian and Micronesian)	13	4.2
	North/South/Central American	2	0.7
	Mixed ethnicity	2	0.7
	Indian	1	0.3
**Employment (*n*** **= 308)**			
	Employee	140	45.5
	Self-employed	33	10.7
	Home duties/caring for children or family	15	4.9
	In education (going to school, university, etc.)	4	1.3
	Doing voluntary work	10	3.3
	Unable to work because of illness	6	2.0
	Unable to work for other reasons	1	0.3
	Retired	99	32.1
**Relationship Status (*n*** **= 309)**			
	Single	39	12.6
	Married/De Facto (living with partner)	230	74.4
	In a relationship but not living with partner	7	2.3
	Divorced/separated	24	7.8
	Widowed	9	2.9

### Weight gain

Weight at diagnosis was reported by 90% of respondents (278 women) and current weight was reported by 95% of respondents (293 women). The proportion of women who were overweight or obese (BMI > 25) increased from 48% at the time of diagnosis, to 67% at the time of completing the survey. In particular, the proportion of women who were obese almost doubled, from 17 to 32%. Mean current and pre-cancer self-reported weight of survey respondents was 76.08 kg (*SD* = 15.49, range, 46–150 kg) and 71.24 kg (SD 14.01, range 47–158) respectively. Mean self-reported current BMI was 28.02 (*SD* = 5.88, *n* = 285) and mean pre-cancer BMI was 26.37 (*SD* = 5.92, *n* = 271). One fifth (21.03%) of women went from being in the healthy weight range at diagnosis (BMI < 25), to an unhealthy weight range (BMI > 25), and 60.52% of women reported an increase of BMI of greater than 1 kg/m^2^.

Most women (64%) reported having gained weight overall after diagnosis, with an average weight gain of 9.07 kg in this group. Of the women who reported gaining weight overall, 77.14% of women gained ≥5 kg of weight. Weight gain was not correlated with time since diagnosis (*n* = 173, *r* = .114, *p* = 0.07). More than half (52.85%, *n* = 148/280) of women rated their concern about weight as high (8–10).

### Other medical conditions and symptoms

Table 2 describes the current medical conditions and symptoms that were being experienced by the respondents. The majority (62.19%, *n* = 125/201) of women reported they were currently using hormonal therapy, of which 40% were using tamoxifen, and 44% were using an aromatase inhibitor.

**Table 2:** Medical and lifestyle characteristics of survey respondents

	Description	N	%
**Medical conditions and symptoms (*n*** **= 228)**			
	Diabetes not requiring insulin	11	4.8
	Impaired glucose tolerance (abnormal glucose tolerance test)	10	4.4
	Fasting hyperglycemia (high blood sugar levels but no diabetes)	5	2.2
	High cholesterol	79	34.7
	High blood pressure	78	34.2
	Neuropathy	64	28.1
	Hot flushes	152	66.7
**Self-Rated Diet (*n*** **= 302)**			
	Excellent	24	7.95
	Very good	126	41.72
	Good	118	39.07
	Fair	29	9.60
	Poor	5	1.66
**Smoking Status (n = 302)**			
	Never Smoked	192	63.58
	Ex-Smoker	101	33.44
	Recently Quit, Ex-smoker in the last 3 months	3	0.99
	Current Smoker	6	1.99
**Alcohol (*n*** **= 292)**			
	Non-drinker	76	26.03
	1–7 standard drinks a week	171	58.56
	8–14 standard drinks a week	40	13.70
	> 14 standard drinks a week	5	1.71
**Physical activity level (MET) (*n*** **= 305)**			
	None (< 80)	17	5.57
	Low (80- < 400)	110	36.07
	Moderate (400- < 560)	65	21.31
	High (> = 560)	113	37.05
**Current Physical Activity (c.f before diagnosis) (*n*** **= 294)**			
	I’m more active	70	23.81
	I’m less active	113	38.43
	I’m as active as I was	111	37.76
**Self-Rated Health (*n*** **= 292)**			
	Excellent	23	7.88
	Very good	100	34.25
	Good	121	41.44
	Fair	45	15.41
	Poor	3	1.02

*MET* metabolic cost (per week) in minutes

### Lifestyle habits

Table 2 details the lifestyle habits of respondents. About 40% of women had tried some kind of diet in the previous 12 months, with the most popular diets being a “healthy balanced” diet (25/124), the 5:2 diet (26/124), vegetarian (17/124), Weight Watchers (17/124), the Dukan and Atkins diets (11 and 7/124 respectively), and meal replacements (5/124). In all, 23 different kinds of diets had been tried. The majority (58.6%) of women reported eating the recommended serves of fruit and vegetables, and 88.8% of women described their diet as excellent (*n* = 24), very good (*n* = 126) or good (*n* = 118). The majority of women (83.6%) rated their health as good and above, although 38.4% of women reported that they were less active than they were at the time of cancer diagnosis and 41.6% did no exercise or low levels of PA. About a quarter of women had been told to restrict their diet. Of these women, 10/55 reported being told to stop eating dairy, whilst eliminating red meat (9%, *n* = 5) and reducing volume/portion size (9%, *n* = 5) was also commonly given advice, mostly by an oncologist or a nurse.

**Fig. 1:**
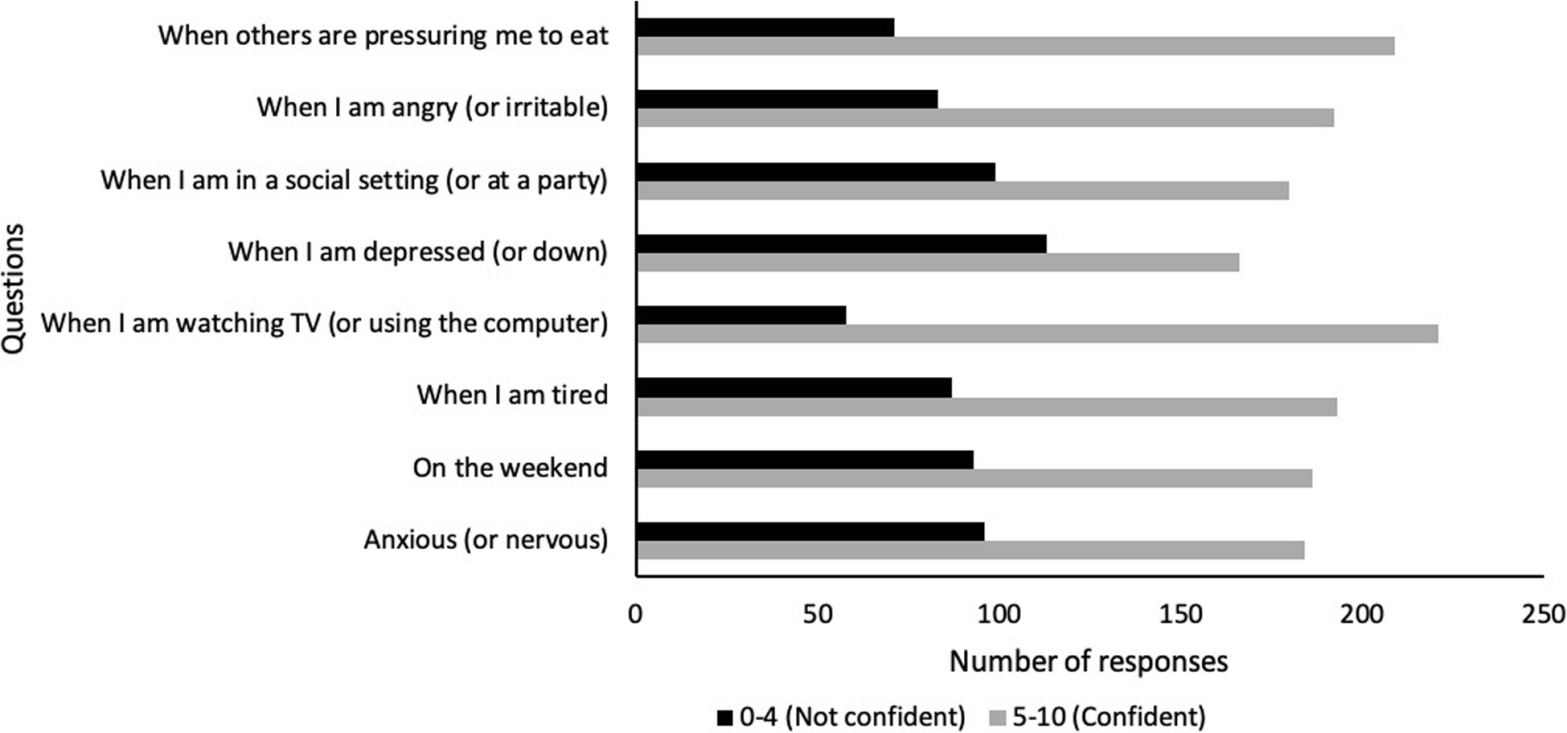
Responses to individual WEL-SF questions

The total number of respondents varied across the WEL-SF questions from 275 to 280. The majority of women rated themselves as moderately to very confident across all questions although they were slightly less likely to rate themselves as confident (0–4) for the questions on resisting eating when depressed and down (40.5%, *n* = 113), and when in a social setting (36%, *n* = 99) (see Fig. 1).

### Advice about weight loss or weight gain

More than three quarters (79.79%, *n* = 233/292) of women reported not receiving any advice about weight loss or weight gain prevention at the time of diagnosis. If advice was given, it was provided mostly by an oncologist (46%, *n* = 26/56) or a BC nurse (12.5%, *n* = 7/56).

### Treatments for weight loss

**Fig. 2:**
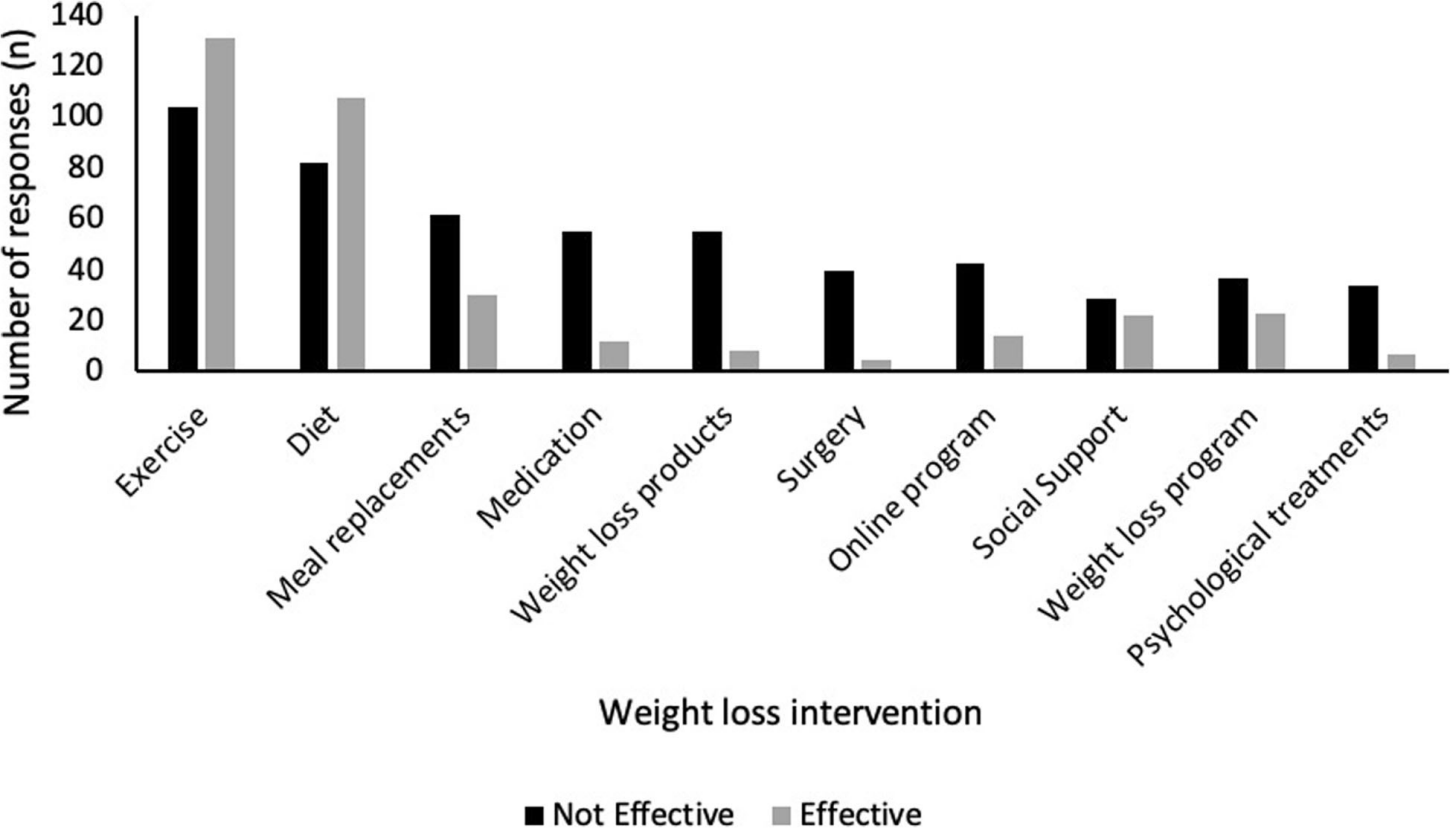
Perceived effectiveness of weight loss interventions

Figure 2 details the number of responses for each of the treatments in terms of their perceived effectiveness for weight loss. Overall, there were few weight loss treatments that women felt were moderately to extremely effective (3–5) including exercise (*n* = 131) and diet (*n* = 108).

### Barriers to weight loss

**Fig. 3:**
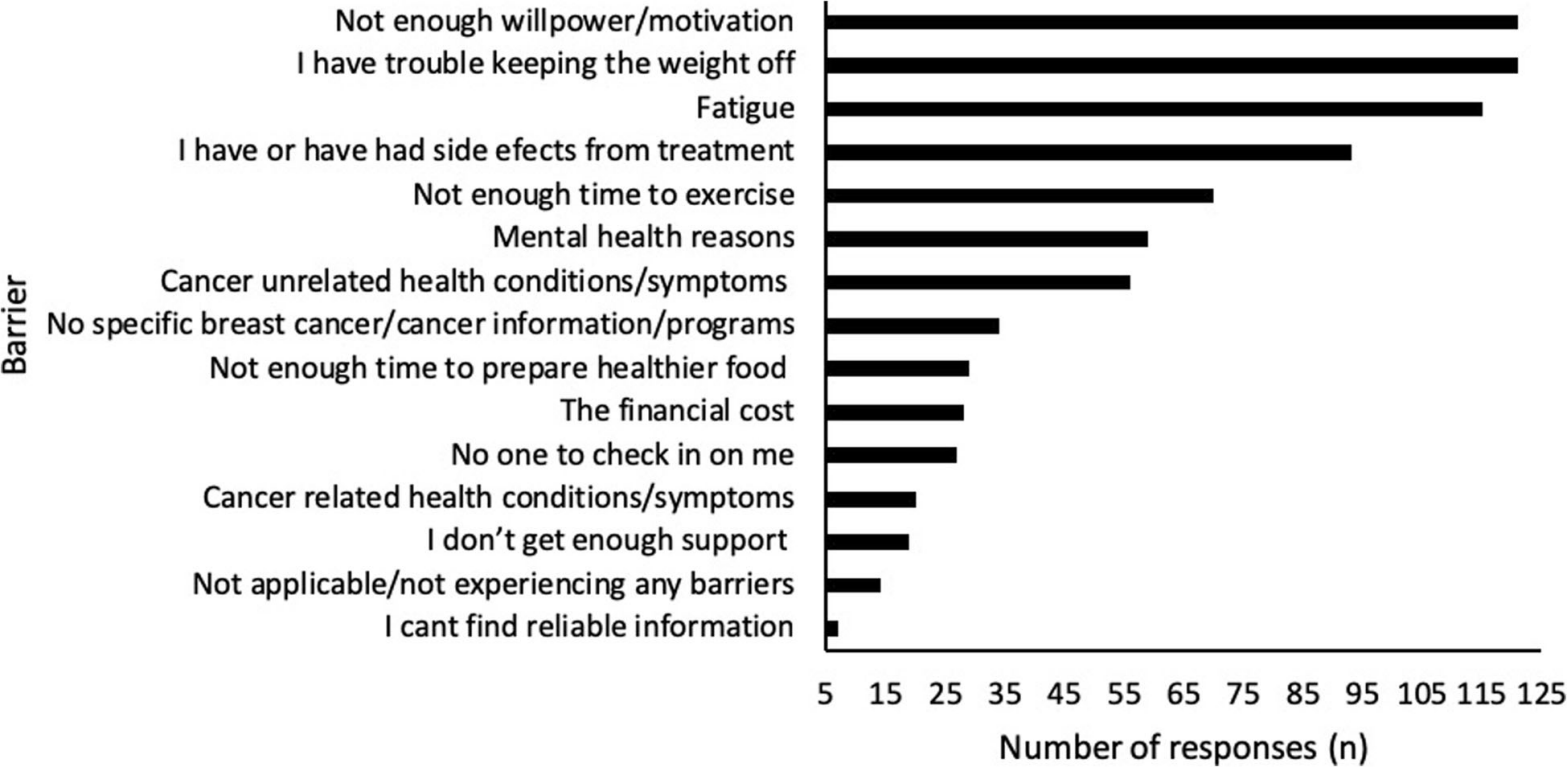
Perceived barriers to weight loss

Figure 3 describes the perceived barriers to weight loss in this cohort of women (*n* = 256).

Women who reported experiencing hot flushes were 2.53 times more likely to report fatigue as a barrier (95% CI 1.53–4.19, *p* = 0.0001) while the relationship between peripheral neuropathy or lymphoedema and fatigue was not significant. The relationship between willpower and fatigue as cited barriers approached statistical significance (OR 1.58, 95% CI .96–2.60, *p* = 0.0547).

Women who cited fatigue as a barrier were almost twice as likely to be doing low levels of PA or no PA than women who did not cite fatigue as a barrier (OR 1.86, 95% CI 1.12 3.08, *p* = 0.0107). However, there was no association between experiencing hot flushes and doing low or no levels of PA.

### Facilitators of weight loss

**Fig. 4:**
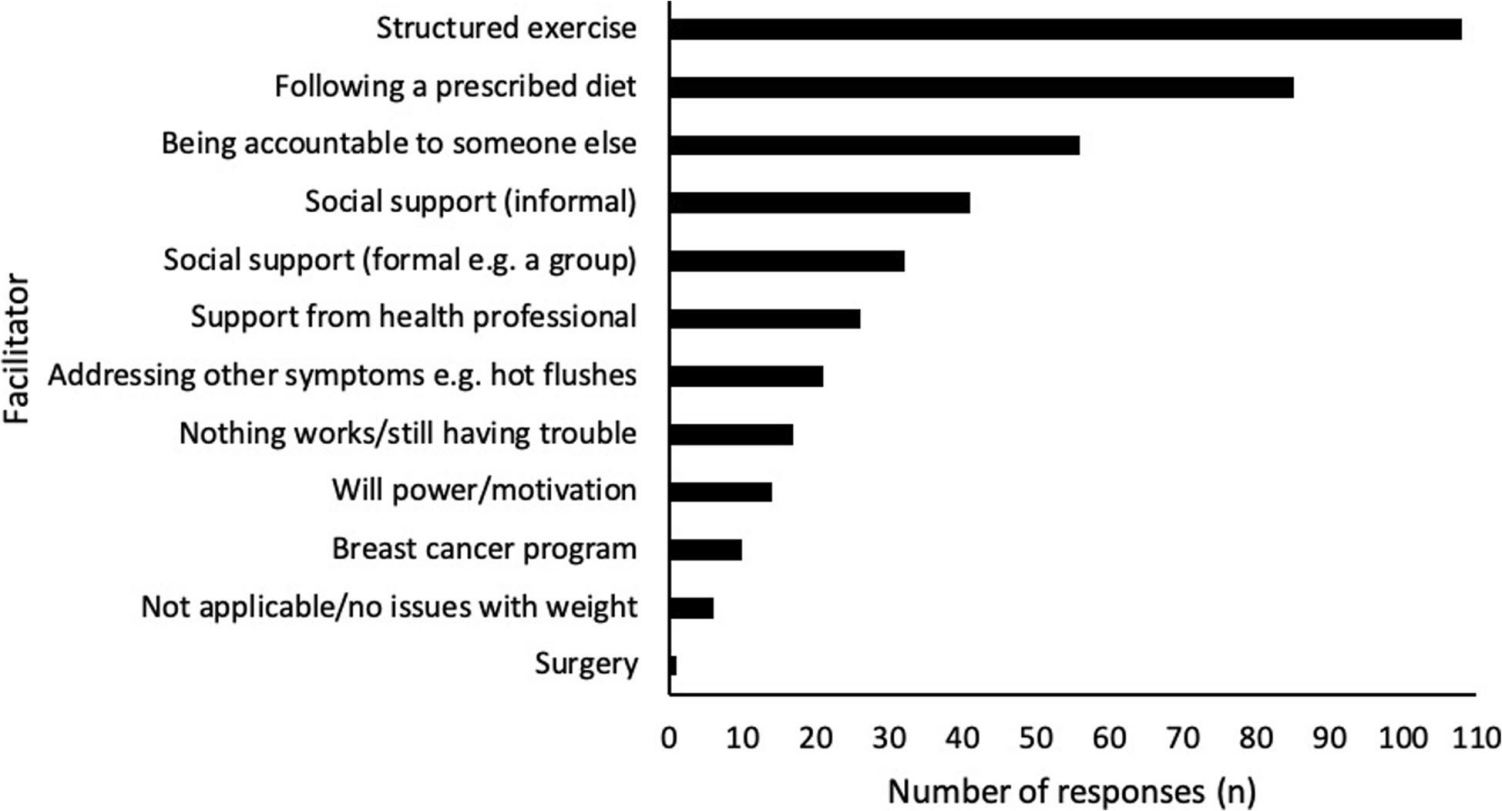
Perceived facilitators of weight loss

Figure 4 describes the perceived facilitators of weight loss in this cohort of women (*n* = 233). The most commonly described facilitators were a structured exercise program (46.4%, *n* = 108), prescribed diet (36.5%, *n* = 85), accountability to someone else (24.0%, *n* = 56) and social support (17.6%, *n* = 41). Only 4.3% (*n* = 10) of women thought a breast cancer specific program would be helpful.

### Research priorities

Among 273 respondents to the question on research priorities, the following were prioritised: PA (68.1%, *n* = 186), weight maintenance (56.0%, *n* = 153), diet (53.1%, *n* = 145), and social support programs (39.6%, *n* = 108). Few women wanted more research on surgical treatments (5.86%, *n* = 16), psychological strategies (5.13%, *n* = 14) or individualised programs (1.1%, *n* = 3).

## Discussion

In this survey of Australian women with breast cancer, we report an increase in the proportion of overweight and obese women from time of diagnosis to post diagnosis, high levels of concern about weight gain, and significant gaps in service provision around weight management and weight gain prevention.

Less than one quarter of women reported receiving advice about weight loss or weight gain prevention at the time of diagnosis. Findings from surveys of oncologists in Canada and the UK are consistent with this data, showing that less than half discuss PA and weight management with their cancer patients [15, 16]. Further, at the time of BC diagnosis women may be more motivated and receptive to lifestyle change [17] suggesting a missed opportunity for health professionals to provide reliable recommendations for lifestyle and weight management to BC patients. Advice on the importance of weight gain prevention should be incorporated into standard breast cancer management advice in order to optimize outcomes for BC survivors. Additionally, the most commonly visited health care providers were reportedly breast surgeons, physiotherapists and medical oncologists. These health professionals could play a vital role in monitoring weight, providing advice on weight gain prevention and referring to a multidisciplinary team. In particular, exercise physiologists and dieticians can play an important role in tailoring diet and exercise interventions for the individual woman.

Although the majority of women described their diet as excellent, very good or good, with 57% reportedly consuming the recommended daily intake of fruits and vegetables, and most women reporting moderate to high levels of weight self-efficacy, women reported generally high levels of concern about their weight. Of concern, 15% of women were drinking more than the recommended intake of one standard drink per day for BC survivors, which may place them at increased risk of BC recurrence [18]. This reveals a gap between a perceived healthy diet and difficulty managing weight, with the need for additional support for women after BC diagnosis.

A small number of women had been advised to avoid red meat and dairy by their healthcare providers. A meta-analysis of 22 prospective cohort and five case control studies found that high and modest dairy consumption significantly reduced the risk of breast cancer compared with low dairy consumption [19]. In particular, yogurt and low-fat dairy reduced the risk of breast cancer while other dairy product types did not. As for red meat, a meta-analysis of 18 studies (a mix of cohort, nested case-control and randomised controlled trials) reported a 6% increase in BC risk (pooled RR 1.06) when comparing the highest to lowest category of unprocessed red meat consumptions, with a higher increased risk for processed red meat consumption of 9% [20]. This suggests that dietary advice for women with BC needs to be strengthened in order to reflect the current best available evidence.

Physical activity (PA) has multiple benefits on improving physical function, psychological distress, fatigue and quality of life, and may reduce co-morbidity and risk of other cancers as well as possibly improve cancer-specific and all-cause mortality [21]. As per the recent Clinical Oncology Society of Australia position statement on exercise in cancer care, which reflects guidelines produced internationally, people with cancer should be referred to accredited exercise physiologists to assist with progression towards PA goals. However, a significant proportion (38%) of women in our study reported that they were less active than they were before diagnosis, with 41% of women reporting none or low levels of PA, highlighting a gap in meeting the needs of women to achieve adequate PA levels.

Studies have reported that common barriers to health behaviors among BC survivors include higher-level barriers such as not having anyone to exercise with, low social support, and having responsibilities at home, along with individual-level barriers such as lack of willpower and fatigue [22] . Other studies have reported lack of support from family and conflicting advice from health professionals as barriers to healthy eating [10]. Cho et al. conducted a multilevel analysis of barriers to healthy behaviors amongst 97 BC survivors, and reported that most participants cited at least one barrier at the individual level - commonly, physical injury or symptoms (including fatigue), lack of time, and lack of motivation. Family and social obligations were also cited as barriers although less often. One quarter of participants reported at least one barrier at the organizational/environmental level (e.g. a busy job) [22]. These studies are consistent with the cited barriers in our study, of which a lack of willpower/motivation was the most cited, closely followed by difficulty keeping weight off, fatigue, and side effects from treatment. Not surprisingly, women who cited fatigue as a barrier were more likely to report low levels of PA. This is consistent with previous research suggesting that fatigue [23] is a common barrier to PA in young BC survivors. Fatigue is a common symptom in cancer survivors [24], and indeed PA is an effective treatment for post-cancer fatigue especially if supervised [25, 26]. BC survivors who are experiencing fatigue should have access to a holistic and comprehensive approach to management of fatigue including PA supervised by an exercise physiologist, cognitive and behavioural strategies, and mindfulness and yoga-based interventions which show promise in alleviating post-BC fatigue [27, 28]. Our findings also suggest a gap in translation of the evidence on exercise as a treatment for post-cancer fatigue with women who cited fatigue as a barrier to PA possibly not being referred to exercise physiologists, which might be derived from their lower self-reported PA levels. Additionally, only 4% of women thought that a breast-cancer specific program would be helpful. This may be because perceived health stigma is common among people with breast cancer and is associated with negative emotions and reduced health-seeking behaviours [29], and our survey respondents may prefer to avoid being labelled a breast cancer survivor [30].

The most commonly cited facilitator of weight loss was a structured exercise regimen. Other facilitators included following a prescribed diet, being accountable to someone else and informal social support. These correlate well with the research priorities of PA, weight maintenance, diet, and social support programs identified by our respondents. Similar priorities have been identified by breast cancer researchers who acknowledge the difficulty in establishing large prospective randomised trials of physical and dietary interventions after breast cancer [31]. These findings were similar to a study of 14 BC survivors who identified facilitators of weight management as family support, accountability to a coach, habitual PA and dietary changes such as reducing energy intake, increasing vegetable intake and portion control [10]. Overall, only diet and exercise were perceived to be effective for weight loss. The literature supports the effectiveness of this combined approach of diet, PA and behaviour modification. A systematic review on weight loss interventions in women with BC found that most of the interventions addressing a combination of diet, PA and behavior modification (5/8) achieved mean within-group weight losses of 5% or more from baseline, and was associated with 30–40% reductions in insulin and leptin in women after BC treatment [32]. Interventions that treated diet and PA separately and focused less on behavior modification achieved less weight loss [32]. Interventions that used behaviour change techniques such as goal setting and action planning were more effective than those that did not, according to a review of 27 studies [33]. Previous research suggests a 25–50% [34] relative improvement in outcomes from lifestyle changes, however this data is largely from observational studies or poorly designed randomized trials which could reflect bias and/or confounding [35]. Behavioural modification would also be beneficial for the most commonly cited barrier in our survey, “not having enough willpower/motivation”.

We achieved a higher than usual response rate (15%) from the BCNA Survey and Review Group, where the typical response rate is 10% (email communication, Research and Evaluation Manager, BCNA 3 Oct 2017). We also obtained responses across Australia, with the proportion of respondents from each Australian State and Territory being similar to national averages on breast cancer incidence sourced from the Australian Institute of Health and Welfare cancer data [36].

There are some limitations to this study. First, although we achieved a 50% higher response rate from the BCNA Review and Survey Group than what is typically seen, the validity of our findings may be limited by the fact that the Review and Survey Group represents only a small proportion of all BCNA members. Furthermore, all data was self-reported, including diet and PA levels. Self-reported PA levels have low-moderate correlation with direct measurement [37] and memory-based dietary measures, even when more robust than our simple question about fruit and vegetable intake, are considered inaccurate when compared to direct quantification [38]. We did not capture sedentary behaviour nor measures of body composition such as percentage of fat-free mass. However, self-reported surveys allow for ease of data collection, and in this case facilitated a nation-wide survey. Further analysis of factors that predicted self-reported weight gain in our sample will be conducted.

## Conclusion

Women in our study reported gaps in information provision and service provision in terms of weight gain prevention after BC, which is a crucial part of improving outcomes after BC. More research is required on the effectiveness of diet and exercise interventions in BC survivors, particularly with regard to weight gain prevention. Successful weight gain prevention or weight loss programs should incorporate structured PA and dietary changes in combination with behavioural change and social support, and address perceived barriers to weight loss such as symptoms from breast cancer treatment and fatigue.

## Supplementary information

**Additional file 1.**

## Data Availability

The datasets used and/or analysed during the current study are available from the corresponding author on reasonable request.

## Abbreviations

BC: Breast Cancer
BCNA: Breast Cancer Network Australia
BMI: Body Mass Index
DCIS: Ductal Carcinoma In Situ
PA: Physical activity
WEL-SF: Weight Efficacy Lifestyle Scale (Short Form)
