# Supplementary material for: Weight management barriers and facilitators after breast cancer in Australian women: a national survey
Source: BMC Womens Health. 2020 Jul 6;20:140. doi: 10.1186/s12905-020-01002-9 (PMC7336491; doi:10.1186/s12905-020-01002-9)
Supplement: Supplementary file 1 — Additional file 1. [file 12905_2020_1002_MOESM1_ESM.docx]

## Appendix 1 Specific demographic, medical, menopausal and lymphoedema details requested in the survey

## Demographic characteristics

State of residence, highest level of education, ethnicity, employment status, relationship status, current age and age at diagnosis were included to describe the characteristics of women.

## Medical details

Women were asked about their diagnosis, treatments received including treatments received to the axilla, the number of lymph nodes removed, whether they had a reconstruction, use of hormonal treatments, menopausal state (at diagnosis and current), presence of other medical conditions and symptoms such as hot flushes and the presence and severity of lymphoedema.

Women were asked to describe the type of breast cancer they were diagnosed with as either "ductal cancer in-situ (DCIS)", "localised stage breast cancer (where your breast cancer is contained within your breast and/or lymph nodes), "metastatic breast cancer (breast cancer that has spread beyond the breast tissue and lymph nodes to distant parts of the body, such as the bones, liver and lungs; also called advanced, secondary or stage four)" or "inflammatory breast cancer". For convenience, inflammatory breast cancer and metastatic breast cancer were then combined and referred to as advanced breast cancer. Women were also asked to indicate the treatments they received such as “Lumpectomy alone”, “Lumpectomy and radiation”, “mastectomy alone”, “mastectomy and radiation”, “removal of lymph nodes”, “chemotherapy”, “hormonal therapy”, “targeted therapy (Herceptin)”, and “other”. As chemotherapy is invariably not provided to women with DCIS, we recoded the diagnosis as "localised" if a woman indicated that she had received chemotherapy.

Menopausal state at the time of diagnosis was assessed as either “Premenopausal (regular periods with no menopausal symptoms such as hot flushes)”, “Perimenopausal/in the menopausal transition (no periods for at least 2 months, plus hot flushes)”, “Postmenopausal (no periods for at least 12 months)” or “Previous surgical menopausal (both ovaries or uterus/womb had been removed).” Participants who indicated they were premenopausal or perimenopausal at the time of diagnosis were asked if they were having periods before breast cancer treatment and to describe what has happened to their periods now; “they have stopped”, “they stopped and then started again”, “they have become more irregular”, “no change” or “other”.

Lymphoedema severity was defined as either “no problem (no noticeable swelling)”, “mild (soft swelling that is not obvious to others and comes and goes)”, “moderate (swelling with occasional hardness in some areas that is obvious to others and is always present)”, “severe (profuse swelling with thickened skin, constant hardness, and a very large, heavy arm that is extremely obvious to others and is always present) as described elsewhere[7].”

***Lifestyle habits***

Women were asked if they had tried the following specific diets in the previous 12 months: Atkins diet (low carbohydrate), 5:2 diet (eat what you want 5 days a week, send your body into starvation mode for 2 days), Paleolithic diet, Dukan diet (High-protein, low-carb), Vegetarian diet, Vegan diet, Weight Watchers diet, Raw food diet, Ultra low-fat diet, Zon diet, Cambridge diet (very low calories), South Beach diet (low-GI), Other. They were asked if they ate at least the recommended serves of fruit and vegetables a day (2 fruit, five vegetable) with answer options of Yes/No. Self-perceived diet quality was assessed as Excellent/Very Good/Good/Fair/Poor. Smoking was assessed as current cigarrete use (Never smoked/Ex smoker/Recently quit ex smoker (smoked in the last 3 months)/Current smoker) and current smokers were asked to indicate the number of cigarettes they smoked each day. Alcohol intake was assessed as Non drinker/1-7 standard drinks a week/8-14 standard drinks a week/>14 standard drinks a week) and a guide to standard drink sizes was provided. The validated Weight Self Efficacy Scale (WEL-SF)[3] was used to evaluate how confident women now felt about being able to successfully resist the desire to overeat in eight different situations on an 11-point Likert scale from 0 (not confident at all) to 10 (very confident). We further dichotomised the responses into "Not confident" (0-4) and "Confident" (5-10). Physical activity levels were calculated according to the number of 20-minute sessions of less vigorous exercise or more vigorous exercise a week, given a weighting and described in terms of MET (metabolic cost) minutes where MET minutes less than 80 were coded as no physical activity, 80 to 400 as low, 400 to 560 as moderate and more than 560 as high. A value of 4 METs was given to moderate physical activity and 7.5 to vigorous physical activity[26].

*Weight management*

Experiences with a range of weight loss interventions (Exercise, Diet – various: Intermittent fasting, etc (please specify), Meal replacements e.g. shakes, Medication, Weight loss supplements/products, Surgery (please specify), Online program e.g. 12 week Body Transformation, Social support , Weight loss program e.g. Jenny Craig, Psychological treatments such as CBT (Cognitive Behavioural Therapy) and the perceived effectiveness of the interventions on was described using a five-point Likert scale from 1 (not at all effective) to 5 (very effective). The responses were further dichotomized into 1 to 2 (not effective) and 3 to 5 (effective). Women were also asked about perceived barriers and facilitators to successful weight loss and weight maintenance, and what they believed should be research priorities in this area.
